# New York Academy of Medicine—Section on Orthopædic Surgery

**Published:** 1897-06

**Authors:** 


### New York Academy of Medicine—Section in Orthopedic Surgery.

MEETING OF APRIL 16, 1897.

THE NON-CUTTING OR UNBLOODY OPERATION OF LORENZ FOR THE RE-POSITION OF CONGENITAL DISLOCATION OF THE HIP.—This paper, by Dr. G. R. Elliott was chiefly a description of the different steps of the procedure, viz.: 1. The reduction, or bringing the head to the level of the acetabulum. 2. Re-position, or wedging the head into the acetabulum. 3. The formation of a solid acetabulum by manipulation and allowing the child to walk about with the thigh fixed by plaster of Paris at about ninety degrees of abduction. The three steps of the operation were performed under chloroform on a patient, a boy twenty-two months old, by Dr. Elliott, before the members of the Section.

Dr. T. H. Myers reported the successful performance on a similar patient, three and a half years old, of Paci's method of manipulation, viz.: forced extension, flexion and then strong traction downward. There were telescoping, lordosis and all the other signs of dislocation, and one-half inch of shortening. A good deal of force was used in order to cause inflammatory adhesion. The limb was immobilized at thirty degrees of abduction, the spica was changed several times in the following six months, and the girl was then allowed to go about with a walking brace and a high shoe on the sound side. She walked with a splint walk when the appartus was removed. The limbs remain at a nearly equal length.

Dr. W. R. Townsend said it would be a great advance if these cases could be cured without a cutting operation. In his experience and observation open methods had proved unsatisfactory. The patients continue to walk lame, and dislocation is liable to recur. He thought the superiority of the new methods could not be taken for granted at once. He had treated one patient by the Paci method.

Dr. R. H. Sayre had seen but one patient in whom the hip could be distinctly reduced by Bigelow's manipulation, but he had not been allowed to operate. In this case it was necessary to abduct the limb much more than had been done in the patient treated this evening. He had not achieved brilliant success by operating. In one case, after the child had been walking for six months, an abscess developed in or near the joint.

Dr. R. Whitman had operated four times by Lorenz's method and had seen great advantage from the application of twenty-five or thirty pounds of traction for three weeks before the reduction. It facilitates bringing the head down to the level of the acetabulum which at times requires a great deal of force.

Dr. H. L. Taylor also thought these patients should have extreme forcible traction before the operation, in order to overcome more thoroughly the muscular contraction. Operative treatment had not been so far very encouraging,and he believed that this procedure held out a good prospect.

Dr. Whitman said that a point in its favor was that mothers would consent to it when they would not consent to a cutting operation. Moreover it did not confine the patient in a hospital or even in bed.

Dr. Elliott said Paci's and Lorenz's procedures were entirely distinct. Paci aimed to build up a nearthrosis in the vicinity of the joint. Frequently the head did not pass the acetabulum. His manipulations were first flexion to the physiological limit, then abduction, then lateral rotation and slow extension, then plaster of Paris for three months, and then careful walking with an apparatus. In this original procedure of Lorenz, however, if entrance of the head was not obtained he deemed the case a failure. It was this re-position plus loading the weight of the body on the bone which made the operation. The acetabulum was there, but rudimentary. The parts immediately began to develope when the bone was replaced. The presence of the bone stimulated the growth which had been absent. The force required in traction was sometimes very great.

### ACHILLO—BURSITIS ANTERIOR.

In a paper on this subject, Dr. S. Lloyd stated that the affection was the result of traumatism from tight shoes, shoes wearing the heel, bicycle riding, jumping and fractures; or the result of septic, tubercular, gonorrhœal or rheumatic infection. The symptoms were pain under the tendo-achillis on standing and walking and in the plantar region, swelling on the outer side of the tendon, hyperidrosis and extensive inflammation of the surrounding tissues. In the treatment cold and warm baths, the application of tincture of iodine and mercurial inunctions were useless. Traumatic cases required prolonged rest and pressure, and cases having their origin in infection should be treated by incision curetting and drainage.

Dr. Whitman presented a case of this affection in a woman of thirty-five years, who was on her feet from six a. m. till eight p. m. The symptoms, of one month's duration, had been pain in the heel and in the metatarsal joints and on pressure of the os calcis. There was slight thickening. He said these cases often became chronic and acquired weakness of the arch, or flat-foot. Rest should be enforced. Acute cases required a plaster of Paris bandage, and chronic ones a brace to arrest the function of the joint.

Dr. Sayre had seen cases among athletes, especially hurdle races, who in making a leap landed on their toes.

Dr. Myers had personally suffered an attack of this kind after a long bicycle ride. He could only walk easy by everting the foot. In plaster, the foot should be placed at right angles to prevent the trouble from becoming chronic.

Dr. V. P. Gibney said that before the pathology was clear these cases used to be called rheumatism of the heel. The region of the tendo Achillis had not been clinically explored. A counterpart is found in the advance in our knowledge which enables us to recognize scurvy in the swelling of joints in children who were called rheumatic.

